# Combining CDKN1A gene expression and genome-wide SNPs in a twin cohort to gain insight into the heritability of individual radiosensitivity

**DOI:** 10.1007/s10142-019-00658-3

**Published:** 2019-01-31

**Authors:** Joanna Zyla, Sylwia Kabacik, Grainne O’Brien, Salma Wakil, Najla Al-Harbi, Jaakko Kaprio, Christophe Badie, Joanna Polanska, Ghazi Alsbeih

**Affiliations:** 1 Data Mining Division, Faculty of Automatic Control, Electronic and Computer Science, Silesian University of Technology, Akademicka 16, 44-100 Gliwice, Poland; 2 Cellular Biology Group, Radiation Effects Department, Centre for Radiation, Chemical and Environmental Hazards, Public Health England, Chilton, Didcot, OX11 0RQ UK; 3 Cancer Mechanisms and Biomarkers Group, Radiation Effects Department, Centre for Radiation, Chemical and Environmental Hazards, Public Health England, Chilton, Didcot, OX11 0RQ UK; 4 Department of Genetics, King Faisal Specialist Hospital and Research Centre, Riyadh, 11211 Kingdom of Saudi Arabia; 5 Radiation Biology Section, Biomedical Physics Department, King Faisal Specialist Hospital and Research Centre, Riyadh, 11211 Kingdom of Saudi Arabia; 6 Department of Public Health and Institute for Molecular Medicine FIMM, University of Helsinki, 00140 Helsinki, Finland

**Keywords:** Radiation response, CDKN1A, *p* value integration, Twin study, GWAS

## Abstract

Individual variability in response to radiation exposure is recognised and has often been reported as important in treatment planning. Despite many efforts to identify biomarkers allowing the identification of radiation sensitive patients, it is not yet possible to distinguish them with certainty before the beginning of the radiotherapy treatment. A comprehensive analysis of genome-wide single-nucleotide polymorphisms (SNPs) and a transcriptional response to ionising radiation exposure in twins have the potential to identify such an individual. In the present work, we investigated SNP profile and *CDKN1A* gene expression in blood T lymphocytes from 130 healthy Caucasians with a complex level of individual kinship (unrelated, mono- or dizygotic twins). It was found that genetic variation accounts for 66% (95% CI 37–82%) of *CDKN1A* transcriptional response to radiation exposure. We developed a novel integrative multi-kinship strategy allowing investigating the role of genome-wide polymorphisms in transcriptomic radiation response, and it revealed that rs205543 (*ETV6* gene), rs2287505 and rs1263612 (*KLF7* gene) are significantly associated with *CDKN1A* expression level. The functional analysis revealed that rs6974232 (*RPA3* gene), involved in mismatch repair (*p* value = 9.68e−04) as well as in RNA repair (*p* value = 1.4e−03) might have an important role in that process. Two missense polymorphisms with possible deleterious effect in humans were identified: rs1133833 (*AKIP1* gene) and rs17362588 (*CCDC141* gene). In summary, the data presented here support the validity of this novel integrative data analysis strategy to provide insights into the identification of SNPs potentially influencing radiation sensitivity. Further investigations in radiation response research at the genomic level should be therefore continued to confirm these findings.

**Electronic supplementary material:**

The online version of this article (10.1007/s10142-019-00658-3) contains supplementary material, which is available to authorized users.

## Introduction

Radiation therapy is a leading modality for cancer treatment. Although continuous technological improvements result in amelioration of radiotherapy protocols leading to precise tumour localisation and better dose delivery accuracy, patient inter-individual response to ionising radiation (IR) exposure is still a considerable risk factor (Pajic et al. 2015). Most patients do not present early, or late, normal tissue toxicity following radiotherapy and they are considered to be radioresistant. But a minority of patients develop severe complications during the course or at the end of the treatment, like skin erythema, nausea, diarrhoea and many others, after receiving a relatively low cumulative dose of radiation (Badie et al. 1995b; Lobachevsky et al. 2016). They are classified as radiosensitive. High-energy X-rays delivered to the cells cause water radiolysis and thereby production of reactive oxygen species (ROS) which indirectly damage DNA (Mettler 2012). The direct interaction between radiation and DNA leads to a range of DNA damage. Amongst them, double-strand breaks (DSBs) are the most toxic to the cells, leading to cell death or permanent cell cycle arrest if unrepaired. Therefore, efforts should be made to improve knowledge and identification of individuals sensitive to ionising radiation to improve radiation therapy efficiency and radiation protection (West and Barnett 2011). Individual radiosensitivity can be influenced by many factors such as DNA damage signalling and DNA repair (Vignard et al. 2013; Badie et al. 1995a, 1997; Morgan and Lawrence 2015), epigenetic modifications (Antwih et al. 2013) or genomic sequence variation (Curwen et al. 2010; Finnon et al. 2008). Some genes, mostly participating in DNA double-strand break repair process, were identified to be involved in human radiosensitivity, e.g. *ATM*, *LIG4* and *PRKDC* (West and Barnett 2011). In this study, we focus on the expression *CDKN1A* (cyclin-dependent kinase inhibitor-1A) which encodes p21 protein and is regulated by p53 protein involved in cell cycle regulation and arrest following DNA damage (Cazzalini et al. 2010; Chen et al. 2015a; Galluzzi et al. 2016). *CDKN1A* also plays a crucial role in various cancer development (Abbas and Dutta 2009; Dunlop et al. 2012; Soltani et al. 2017). Several studies show an association between *CDKN1A*-SNPs and cancer and patient survival prognostics (e.g. Cazier et al. 2014; Kang et al. 2015; Vargas-Torres et al. 2016). A recent study of Price et al. (2015) suggests that *CDKN1A* regulates Langerhans cell and could influence the response of cutaneous tumours to radiotherapy. *CDKN1A* abnormal expression has been reported to be associated with acute sensitivity to radiation (Amundson et al. 2003; Badie et al. 2008; Szołtysek et al. 2018). In Alsbeih et al. (2007), they show that individual response in *CDKN1A* is related to inherent radiosensitivity. It is, therefore, assumed that *CDKN1A* expression level might be predictive of radiation toxicity and an investigation that allows explaining inter-patient *CDKN1A* expression variability is of high importance.

Many high-throughput approaches are currently used to gain an understanding of radiosensitivity; amongst them, the analysis of single-nucleotide polymorphisms (SNPs) is one of the most promising to investigate radiation response (Andreassen et al. 2012). Radiogenomics, which concentrates on the relation between genomics and radiation toxicity, has gained a high interest lately (West and Barnett 2011). Although a large number of studies have been reported (e.g. Best et al. 2011; Kerns et al. 2018; Mumbrekar et al. 2016; Rosenstein 2011), there is a need to continue identifying genes and SNPs that affect radiosensitivity to understand better the mechanism underlying radiation toxicity in sensitive patients. The choice of methods for data analysis allowing identification of relevant SNPs depends on the study design. Different statistical approaches have been widely discussed and presented (Bush and Moore 2012; Evangelou and Ioannidis 2013). Twin-based study designs were pointed as a promising source of information in genomics (Andrew et al. 2011; Bataille et al. 2012; Chen et al. 2015b; Tan et al. 2010) and transcriptomics (Majewska et al. 2017; Mamrut et al. 2017). In the following study, a dataset of a complex structure and small sample size with related (dizygotic and monozygotic twins) and unrelated individuals and quantitative measurement of *CDKN1A* gene expression as a metric of radio-toxicity is analysed. Such data structure is rarely studied and requires the development of dedicated signal analysis pipeline supporting the potential identification of a genetic signature of radiosensitivity. A literature screen revealed that a variety of quantitative trait loci (QTL) sib-pairs type methods are proposed to study related individuals (Kruglyak and Lander 1995a; Sham et al. 2002; Visscher and Hopper 2001). Several statistical approaches dedicated to the sample analysis of unrelated individuals are also available. We concluded that there is a lack of simple solutions available which would apply to complex study designs.

To fill that gap, we propose a novel signal analysis pipeline combining classical biometrical models (Kruglyak and Lander 1995b) and cross-sample *p* value integration methods. Although challenging, the integration approach appears to be the most promising methods in genome-wide studies (Moore et al. 2010; Stranger et al. 2011). The origin of integration methods arose from meta-analyses, where meta-genome-wide association studies (GWAS) brought new light to specific diseases (Barrett et al. 2009; Pharoah et al. 2013). Statistical integration in GWAS and SNP identification was previously presented as one of the most promising ways of analysis (Chen 2013; Chen et al. 2014; Zaykin and Kozbur 2010). In this study, we proposed to use statistical integration across individuals of different kinship for the validation of SNPs associated with radiation response. We demonstrated that the proposed procedure of integration improved the statistical analysis, especially in the case of small sample size studies. Finally, new promising candidate polymorphisms describing the association between genomics and radiation response in healthy individuals were identified.

## Material and methods

### Material

T lymphocytes were previously collected from healthy young adults of European ancestry sampled from the Finnish Twin Cohort Study (Finnon et al. 2008). The group under investigation here included 130 individuals divided into three subgroups according to their kinship: (1) 44 unrelated individuals (unR); (2) 28 dizygotic twin pairs (DZ) and (3) 15 monozygotic twin pairs (MZ). *CDKN1A* gene expression was measured for every individual by qPCR technique at two conditions: control (no irradiation (0 Gy)) and 2 h after sample irradiation with a single dose of 2 Gy of X-ray. The irradiation was performed at room temperature with an A.G.O. HS X-ray system by Aldermaston, Reading, UK—output 13 mA, 250 kV peak, 0.5 Gy/min. Detailed information about sample collection, storage and experiment was presented in (Kabacik et al. 2011; Manning et al. 2013). Additionally, DNA was extracted from all control samples using the DNeasy kit (Qiagen) and sent for genotyping. Analysis of 567,096 SNPs was performed by Axiom GW Human hg36.1 arrays (Affymetrix, ThermoFisher Scientific) according to manufacturer’ instruction. The used arrays did not include polymorphisms present in *CDKN1A* gene; thus, only SNPs in genes that interact with *CDKN1A* could be investigated in presented work.

### Methods

#### Data pre-processing

All genotyped SNPs were annotated to the genome version 38 (according to NCBI resources). The standard GWAS specific quality control was performed, including minor allele frequency (MAF) control with level 10% and call rate on 90% (Turner et al. 2011). The quality control procedures reduced the number of SNPs from 567,096 to 383,322 (none of them was located in *CDKN1A*). The internally standardised ratio between the response at 2 Gy and referenced 0 Gy was calculated for investigated biomarker (*CDKN1A*) per each person. The 2 Gy vs 0 Gy ratio value will represent the radiation response of the investigated biomarker.

#### Heritability

At first, the hypothesis of the mean equality between MZ and DZ twin signals of 2 Gy vs 0 Gy ratio of *CDKN1A* expression was tested by a modified *t* test procedure proposed by Christian (1979). Further, the homogeneity of the MZ and DZ intra-class Pearson correlations was tested with the use of *z*-transformation (Fisher 1992). The assessment of genetic heritability of the trait was done by structural equation modelling (SEM) for the variance decomposition method, which bases on standard Falconer’s formula (Falconer 1965; Neale and Cardon 1994). The standard weights for additive (A) and dominant (D) genetic effects were set for monozygotic twins and equalled one for both effects. The 0.5 for additive effect and 0.25 dominant effect were considered for dizygotic twins. Common environment (C) weight values equal to 1 for both DZ and MZ twins as analysed twin pairs were reared together. The ACE and ADE models and all their submodels were constructed with the use of OpenMx (Neale et al. 2016). The Bayesian information criterion (BIC) was applied for model selection (Schwarz 1978). Additionally, the ADE and AE models were tested by a log-likelihood ratio test (LRT) for their over performance of the simple E model. To each model component, its 95% confidence interval (CI) was calculated.

#### Statistical analysis: unrelated

To verify the hypothesis on equality of signal means across observed genotypes, the adequate statistical test was performed on the probe of unrelated individuals (Bush and Moore 2012). The three different models of SNP-*CDKN1A* expression interactions were checked: genotype, dominant and recessive (Lettre et al. 2007; Zyla et al. 2014). Normality of *CDKN1A* expression’s distribution was calculated by the Shapiro-Wilk test, and homogeneity of variances was verified by Bartlett’s test or *F* test. Depending on their results, parametric (ANOVA, *t* test, the Welch test) or non-parametric (the Kruskal-Wallis, Mann-Whitney-Wilcoxon) tests were applied. The best model of SNP-*CDKN1A* interaction was assigned to each SNP based on calculated *p* values with the use of minimum *p* value criterion.

#### Statistical analysis: twin analysis

**Fig. 1:**
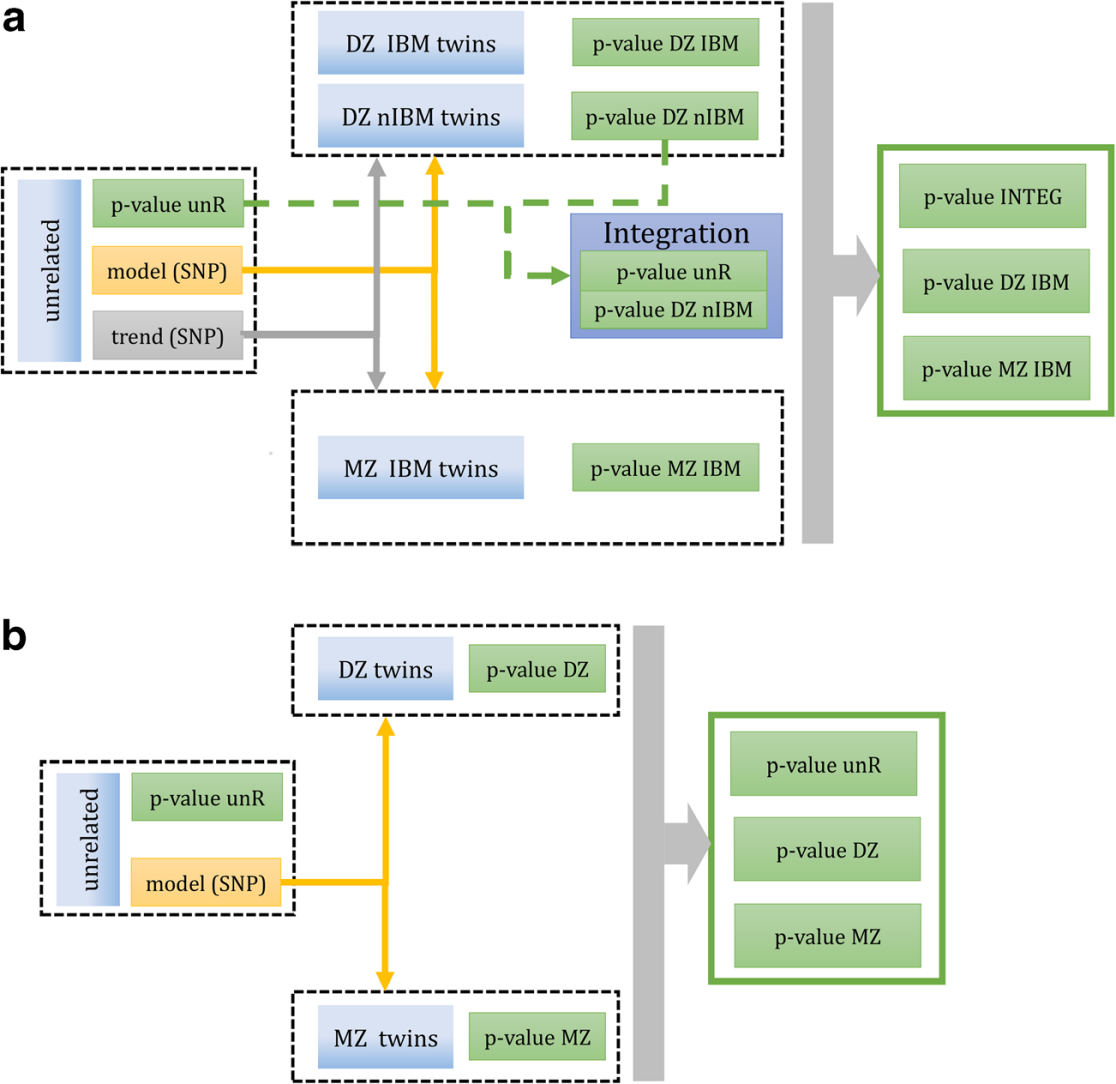
The statistical analysis pipelines, where **a** represents the standard statistical analysis and **b** represents the developed novel statistical analysis procedure. Both are dedicated to the testing association in complex study design

The novelty of presented work is stated for twin analysis. The SNP specific best model of SNP-*CDKN1A* interaction, obtained in the group of unrelated individuals for particular SNP, was used to split twin pairs into two subgroups named as *identical by model* (IBM) and *non-identical by model* (nIBM) following rules presented in Table 1. Splitting was done independently for each SNP; the difference of signal level (2 Gy vs 0 Gy ratio) between twins was calculated. In the case of IBM twins, the hypothesis on the average difference of signal between twins being equal to zero was verified. For nIBM twins, the null and alternative hypotheses depended on signal trend observed amongst unrelated individuals. For example, in the case of a significantly higher level of *CDKN1A* gene expression observed in Ax group vs. BB group in unrelated (unR) population, the same relation was tested in DZ and MZ subgroups by properly formulated one-side tests. The above-described procedure allows for response trend control in the process of signal validation. During the next step, the integration of *p* values from unR and DZ nIBM was performed. In the case of a dominant or recessive model of SNP-*CDKN1A* interaction, weighted *z*-method (Lipták 1958; Mosteller et al. 1954) with an inverse of standard error (1/SE) as the weighting factor was used (Whitlock 2005), while for genotype model, the Lancaster integration procedure was applied (Lancaster 1961). The procedure was not applied to data on monozygotic twins, who have the same genotype; hence, only *identical by model* twins were observed. Polymorphism was considered as associated with *CDKN1A* expression if unR and nIBM DZ combined *p* value was less than 0.001 and there was no evidence to reject the null hypothesis on equality of response between DZ and MZ at significance level *α* equal to 0.001. The diagram of the proposed analysis is presented in Fig. 1b. Finally, the results of integrative procedure were compared to commonly used non-parametric QTL method proposed by Kruglyak and Lander in (Kruglyak and Lander 1995b) including model weights presented in (Kruglyak and Lander 1995a). The Kruglyak and Lander method is the most common approach used in twin and sib-pair analysis, which allow to include models of genetic interactions. The candidate polymorphisms in this approach are selected as follows: *p* value unR less than 0.001, *p* value DZ QTL less than 0.001, *p* value MZ QTL less than 0.001. The diagram of the standard analysis is presented in Fig. 1a.

**Table 1:** The rules of splitting DZ and MZ twins into *identical by model* (IBM) and *non-identical by model* (nIBM) subgroups based on the best model of SNP-*CDKN1A* interaction found in unrelated population (unR). Letters A and B code for the genotyping results, A stands for reference allele, while B for mutant one

Sibling 1	Sibling 2	The best model of interaction in unR population		
		Genotype	Dominant, AA vs xB	Recessive, Ax vs BB
AA	AA	IBM	IBM	IBM
AA	AB	nIBM	nIBM	IBM
AA	BB	nIBM	nIBM	nIBM
AB	AA	nIBM	nIBM	IBM
AB	AB	IBM	IBM	IBM
AB	BB	nIBM	IBM	nIBM
BB	AA	nIBM	nIBM	nIBM
BB	AB	nIBM	IBM	nIBM
BB	BB	IBM	IBM	IBM

#### In silico genomic functional analysis

In silico functional analysis was performed for sets of candidate radiation response relevant SNPs for signal 2 Gy vs 0 Gy level of *CDKN1A* expression and each analysis approach (standard and novel). The genomic location of each candidate SNP was assessed, and the lists of SNPs linked genes were constructed. Using the resources of SIGNOR 2.0 database (Perfetto et al. 2015), the list of genes which directly interact to/with *CDKN1A* was constructed. Additionally, the list of transcription factors (TFs) of *CDKN1A* gene was obtained using TRRUST 2.0 database (Han et al. 2017). Both lists were compared with obtained candidate polymorphisms. Additionally, the overrepresentation analysis of GO terms (biological process only) and KEGG pathways was performed (Falcon and Gentleman 2006; Kanehisa et al. 2016). The deleterious impact to the human organism of each candidate missense SNP was accessed by the PredictSNP algorithm (Bendl et al. 2014). Finally, the literature research was performed using the PubMed resource.

## Results

### Heritability

First, the intra-class correlation coefficients were calculated for both twin types, and hypothesis on MZ twins’ correlation being smaller or equal to DZ twins’ correlation was tested (*H*_0_: *r*_MZ_ ≤ *r*_DZ_). Correlations between twins for 2 Gy vs 0 Gy ratio equals 0.26 (DZ) and 0.77 (MZ) respectively. Within the monozygotic twins, significantly larger correlation than within dizygotic twins is observed (*p* value = 0.0140). It shows significantly larger signal similarity with increased genetic relatedness. Additionally, the equality of means of *CDKN1A* gene expression between DZ and MZ twins was tested. The Christian procedure brings no evidence against the hypothesis on equality of signal mean values (*p* value = 0.3333). Both outcomes allow for further investigation of heritability. The correlation coefficient for MZ is twice larger than for DZ, which determines the ADE model (and its submodels) to be only considered. The ADE model and its submodels where constructed, and the BIC method was used for model selection. The AE model shows the lowest BIC value and estimates the narrow-sense heritability estimate *CDKN1A* radiation response as equal to 66% (95% CI 37–82%). Detailed results for the main model and submodels are presented in Table 2. All the above support the hypothesis that a large fraction of *CDKN1A* response expression variation is accounted for genetic factors, which is of great importance for further association study.

**Table 2:** Result of heritability investigation for *CDKN1A* expression in response to radiation of dose 2 Gy (2 Gy vs 0 Gy ratio)

Model	BIC	A [95% CI]	D [95% CI]	E [95% CI]	LRT *p* value model vs E model
ADE	240	51 [0–82]	15 [0–81]	34 [0–63]	0.0005
AE	235	66 [37–82]	–	34 [0–62]	0.0001
E	246	–	–	100 [100–100]	–

### Polymorphism investigation

**Fig. 2:**
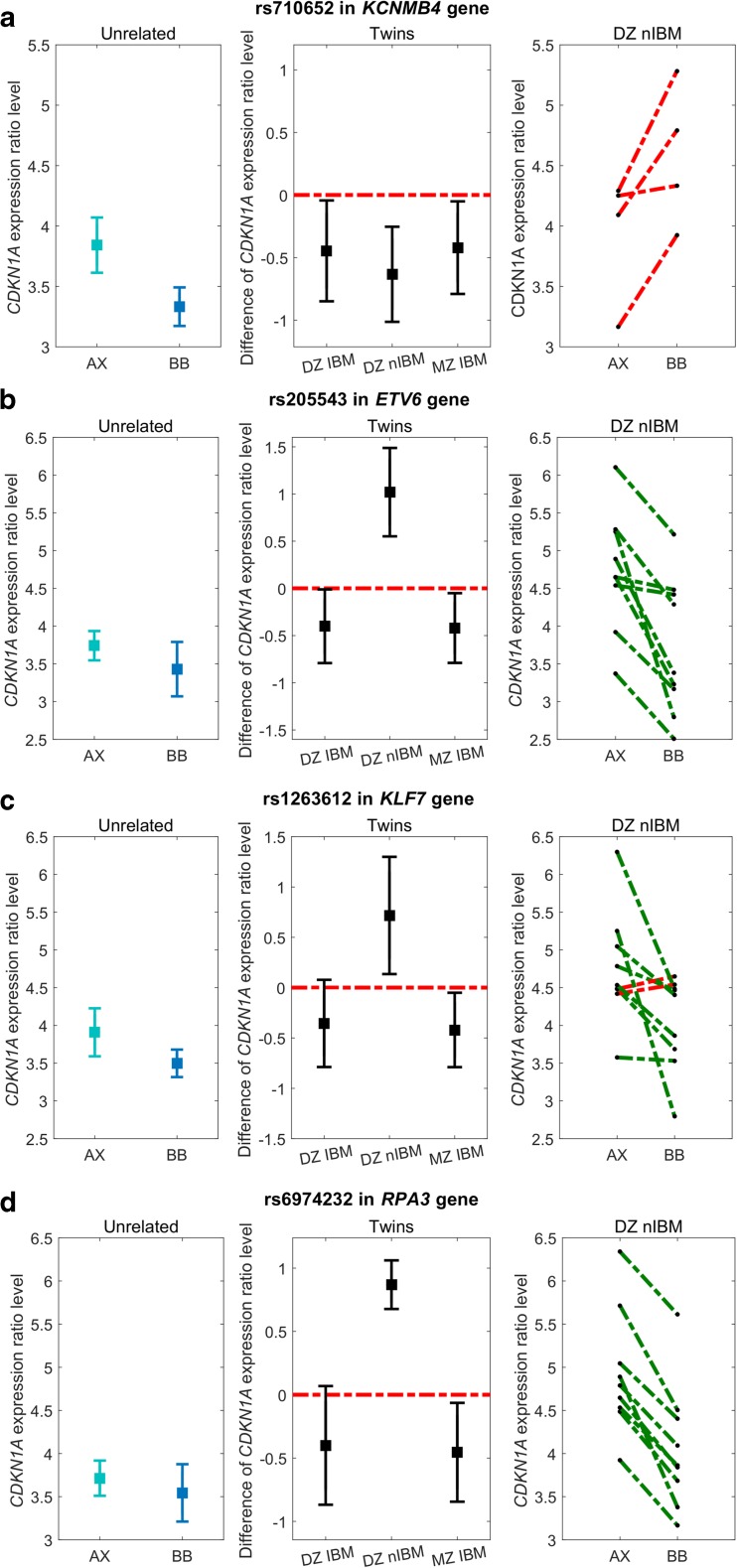
Levels of signal response (2 Gy vs 0 Gy) in the recessive genetic model under different genotypes and different kinship classes for **a** rs710652 polymorphism in *KCNMB4*, **b** rs205543 in *ETV6*, **c** rs1263612 in *KLF7* and **d** rs6974232 in *RPA3* genes. The two left-side plots represent the 95% confidence interval for the mean of *CDKN1A* gene expression. The right-side plots represent the expression levels for non-identical by model (nIBM) dizygotic twins, where discontinued green colour lines represent identical response trend while discontinued red colour lines represent opposite response trend amongst unR and DZ nIBM

The results of the analysis for investigated experimental condition (2 Gy vs 0 Gy ratio) and methods (integrative approach and non-parametric QTL approach as reference) are presented in Table 3. As can be observed, the novel method detects 1804 SNPs, of which 849 are located in transcriptomic regions. Out of all SNPs detected by a novel approach, 81% were also detected via standard approach. Figure 2 panel A presents the exemplary polymorphism detected by the standard approach and not identified by the novel method (rs710652 in *KCNMB4* gene). The first plot (left panel) presents mean *CDKN1A* expression and its 95% CI in the phenotype-genotype interaction model for unrelated individuals. Second plot (middle panel) presents mean value and its 95% CI for expression difference between twin pairs. Third plot (right panel) shows *CDKN1A* expression within DZ nIBM twin pairs and serves as a validation of response trend found in unrelated individuals (left panel). As can be observed, the standard procedure detects polymorphisms, which do not validate by the trend of signal expression observed in unrelated individuals—it can be classified as false discovery. The list of all detected polymorphisms with their genomic information is included in Supplementary Material 1. In the next paragraph, a consideration of the relationship of detected polymorphisms and investigated phenomena is demonstrated.

**Table 3:** The data analysis results (after MZ twin validation) for both methods and signal at 2 Gy vs 0 Gy ratio. The first column represents the standard approach (Stand.), while the second column represents a novel integrative approach (Int.)

2 Gy vs 0 Gy ratio		Genotype		Dominant		Recessive		Total		Common
		Stand	Int	Stand	Int	Stand	Int	Stand	Int	
Initially, # of SNPs		2093		177,481		203,748		383,322		–
*α* = 0.001	# candidate SNPs	1	52	92	839	88	913	181	1804	147 [81%]
	# SNPs in genes	1	25	50	406	45	418	96	849	78 [81%]
	# unique protein-coding genes							81	615	74 [91%]

### In silico functional analysis

A literature study was performed to identify the signalling cascade from the genes with the candidate relevant SNPs to the *CDKN1A* gene. Two types of linkage were studied: (1) interaction, where control of *CDKN1A* expression is done by transcription factor (TFs) or protein phosphorylation with identified SNPs and (2) complex, where a group of genes (with detected SNPs) show an overrepresentation of pathway highly relevant to radiation toxicity. Finally, they are accompanied by missense polymorphism investigation. The list of proteins taking part in direct phosphorylation of p21 (encoded by *CDKN1A*) was obtained using the resources of SIGNOR 2.0 (Perfetto et al. 2015), while TF genes for *CDKN1A* were established via TRRUST 2.0 (Han et al. 2017). From the group of detected polymorphisms, three of them are located in genes responsible for transcription regulation of *CDKN1A* (Table 4). First SNP—rs205543—is located in the *ETV6* gene also known as TEL oncogene. TEL oncogene was shown as TF of *CDKN1A* and *BBC3* and is related to “transcriptional misregulation in cancer” pathway (Yamagata et al. 2006). The rearrangements of *ETV6* were also observed in radiation-associated thyroid cancer (Leeman-Neill et al. 2014). Next, two SNPs (rs2287505 and rs1263612) are located in *KLF7* gene (part of the Kruppel family), which is mainly responsible for cell proliferation, and it transcriptionally regulates *CDKN1A* expression (Smaldone et al. 2004). *CDKN1A* expression level in different kinship subgroups and genotypes for polymorphisms in *ETV6* and *KLF7* genes are presented in Fig. 2. As it can be observed in panels B and C for DZ nIBM twins (middle panel), CIs do not include zero value, which confirms different response caused by different genotypes at a significance level < 0.05. Moreover, the right-side panel plot shows that nIBM dizygotic twins represent the same signal trend as observed in unrelated individuals. For the differences of *CDKN1A* response signal in IBM twin pairs, there is no statistical evidence that it is different from zero. It fulfils the expectation as identical twins that share the same genotype model express the similar *CDKN1A* radiation response. None of the presented polymorphisms was detected by the standard approach.

**Table 4:** Result of the investigation on transcription factors and phosphorylation proteins

Gene	rs ID	Model of interaction	Type of interaction with CDKN1A^a^	Integrated *p* value	Ref
*ETV6*	205543	AX vs BB	TF	4.39e−04	(Yamagata et al. 2006)
*KLF7*	2287505	AX vs BB	RoTL	8.56e−04	(Smaldone et al. 2004)
	1263612	AX vs BB		9.27e−04	

^a^*TF* transcription factor, *RoTL* Regulation on transcription level

Next, the overrepresentation analysis for all obtained genes with SNPs presented in Supplementary Material 1 was performed on KEGG and GO (biological process (BP) only) resources (Table 5). A detailed list is included in Supplementary Material 2. As can be observed, the novel integrative method shows a higher number of overrepresented pathways and GO terms when compared to the standard approach. Out of overrepresented pathways at 2 Gy vs 0 Gy ratio in KEGG and gene ontology (GO) those indicated by *RPA3* gene (with candidate SNPs rs6974232) are highly related to the investigated phenomenon. *RPA3* plays a role in both DNA replication and the cellular response to DNA damage (together with *RPA1* and *RPA2*). In the cellular response to DNA damage, the RPA complex controls DNA repair and DNA damage checkpoint activation (Haring et al. 2008). Recently, Guo et al. showed the relationship between RPA family and distant metastasis in nasopharyngeal carcinoma patients treated with intensity-modulated radiation therapy (Guo et al. 2016). Of the overrepresented KEGG pathways with *RPA3* involvement, we can distinguish mismatch repair (*p* value = 9.68e−04) or DNA replication (*p* value = 2.63e−02). In the case of GO analysis, the following terms were identified: RNA repair (*p* value = 1.4e−03), mismatch repair (*p* value = 1.52e−02) and nucleotide excision repair by DNA gap filling (*p* value = 3.25e−02). As mentioned in the introduction, the DNA repair processes and cell cycle control are crucial for radiosensitivity phenomenon. *RPA3* occurs together with investigated *CDKN1A* in Reactome pathways (Fabregat et al. 2017): mitotic G1-G1/S phases, G1/S transition and cell cycle checkpoints. The response level of *CDKN1A* under different kinship and polymorphism rs6974232 is presented in Fig. 2 panel D.

**Table 5:** The summary results for overrepresentation analysis

	KEGG	GO [BP]
Standard	4	99
Integrative	46	399
Common	4	17

Finally, the missense SNPs were investigated by PredictSNP to assess the possible deleterious impact on protein function. Out of 21 missense polymorphisms, the rs1133833, which change the arginine in position 23 to threonine (R23T) in *AKIP1* gene, was predicted as deleterious with a score of 72%. The *AKIP1* gene encodes A-kinase-interacting protein 1 which regulates the effect of the cAMP-dependent protein kinase signalling pathway on the NF-κB activation cascade. It is well known that IR activates the NF-κB pathway which further makes cancer cell resistant to treatment, while in parallel, the NF-κB has an impact to apoptosis control (Gao et al. 2010; Magné et al. 2006; Molavi Pordanjani and Jalal Hosseinimehr 2016). Additionally, the *AKIP1* is overexpressed in breast cancer and is related to poor prognosis of survival (Mo et al. 2016). Second, a deleterious polymorphism was rs17362588 located in *CCDC141* gene, and it changes arginine in position 935 to tryptophan (R935W; score 87%). The *CCDC141* encodes a coiled-coil domain-containing protein. However, its role is as yet unclear. Several studies show mutations in *CCDC141* in patients with thyroid disorder known as idiopathic hypogonadotropic hypogonadism (Hutchins et al. 2016; Turan et al. 2017). However, in relation to radiation response, apoptosis and *CDKN1A* have not been described in the literature.

## Discussion and conclusions

The work presented here investigated genetic component in *CDKN1A* expression following ionising radiation exposure which was used as a surrogate marker for radiosensitivity of healthy individuals. Firstly, we have shown that *CDKN1A* transcriptional response to radiation is heritable, with a heritability estimate of 66% (95% CI 37−82%) based on a twin analysis. This provided motivation for further investigation at the genomic level (SNP investigation). Additionally, those findings are consistent with previous investigations of heritability for apoptosis and cell cycle delay (Camplejohn et al. 2006; Finnon et al. 2008) and brought new insight of understanding which genes can be responsible for previously observed outcomes. Furthermore, we proposed here a novel signal analysis pipeline for quantitative genomic association analysis of data with different kinship and no family information. The presented workflow is a combination of SNP genotype modelling and statistical integration. It can be an alternative for well-known linkage analysis of sib-pairs, when, in most of the cases, family information is required (Fulker et al. 1999; Li et al. 2005). Additionally, the integration process increases the power of the conducted analysis, which is of great importance when the sample size is small. Finally, the method proposed here includes control of response trends in the process of validation, which allows for reliable candidate polymorphism detection, reducing the number of false positives. The in silico investigation showed that obtained polymorphisms are related to the investigated phenomenon at the global scale via overrepresentation analysis of pathways and gene ontologies. Additionally, the direct interaction with analysed *CDKN1A* expression was shown. SNPs located in CDKN1A transcription factors genes, *ETV6* (rs205543) and *KLF7* (rs2287505, rs1263612), are of special interests for further biological investigation. Further, the rs6974232 in *RPA3* gene should be highlighted as it participates in DNA repair and replication processes which are crucial pathways to radiation response. Finally, the missense polymorphism rs1133833 in *AKIP1* gene with possible deleterious impact to protein function was identified. In summary, the results presented support the validity of the proposed statistical strategy of analysis and demonstrate that high-throughput genomic approaches, such as the one described here, can provide insights to identify radiosensitive patients, and further similar investigations will help to develop future predictive assays for clinical applications.

## Electronic supplementary material

ESM 1 (XLS 614 kb)

ESM 2 (XLS 80 kb)

## Abbreviations

IR: ionising radiation
ROS: reactive oxygen species
DSBs: double-strand breaks
SNP: single-nucleotide polymorphism
QTL: quantitative trait loci
GWAS: genome-wide association study
unR: unRelated
DZ: DiZygotic twins
MZ: MonoZygotic twins
MAF: minor allele frequency
SEM: structural equation modelling
BIC: the Bayesian information criterion
LRT: likelihood ratio test
IBM: identical by model
nIBM: non-identical by model
TF: transcription factor
CI: confidence interval
GO: gene ontology
BP: biological process
KEGG: Kyoto Encyclopedia of Genes and Genomes
